# Topical Drug Delivery in Chronic Rhinosinusitis Patients before and after Sinus Surgery Using Pulsating Aerosols

**DOI:** 10.1371/journal.pone.0074991

**Published:** 2013-09-11

**Authors:** Winfried Möller, Uwe Schuschnig, Gülnaz Celik, Wolfgang Münzing, Peter Bartenstein, Karl Häussinger, Wolfgang G. Kreyling, Martin Knoch, Martin Canis, Sven Becker

**Affiliations:** 1 Institute for Lung Biology and Disease (iLBD), Helmholtz Zentrum München, Neuherberg, Germany; 2 BU Pharma, PARI Pharma GmbH, Gräfelfing, Germany; 3 Department of Nuclear Medicine, LMU Medical Center Grosshadern, München, Germany; 4 Department of Pulmonary Medicine, Asklepios Hospital, München-Gauting, Gauting, Germany; 5 Department for Otolaryngology, University Göttingen Medical Center, Göttingen, Germany; 6 Department of Otolaryngology, Head and Neck Surgery, LMU Medical Center Grosshadern, München, Germany; Hospital of the University of Pennsylvania, United States of America

## Abstract

**Objectives:**

Chronic rhinosinusitis (CRS) is a common chronic disease of the upper airways and has considerable impact on quality of life. Topical delivery of drugs to the paranasal sinuses is challenging, therefore the rate of surgery is high. This study investigates the delivery efficiency of a pulsating aerosol in comparison to a nasal pump spray to the sinuses and the nose in healthy volunteers and in CRS patients before and after sinus surgery.

**Methods:**

^99m^Tc-DTPA pulsating aerosols were applied in eleven CRSsNP patients without nasal polyps before and after sinus surgery. In addition, pulsating aerosols were studied in comparison to nasal pump sprays in eleven healthy volunteers. Total nasal and frontal, maxillary and sphenoidal sinus aerosol deposition and lung penetration were assessed by anterior and lateral planar gamma camera imaging.

**Results:**

In healthy volunteers nasal pump sprays resulted in 100% nasal, non-significant sinus and lung deposition, while pulsating aerosols resulted 61.3+/-8.6% nasal deposition and 38.7% exit the other nostril. 9.7+/-2.0 % of the nasal dose penetrated into maxillary and sphenoidal sinuses. In CRS patients, total nasal deposition was 56.7+/-13.3% and 46.7+/-12.7% before and after sinus surgery, respectively (p<0.01). Accordingly, maxillary and sphenoidal sinus deposition was 4.8+/-2.2% and 8.2+/-3.8% of the nasal dose (p<0.01). Neither in healthy volunteers nor in CRS patients there was significant dose in the frontal sinuses.

**Conclusion:**

In contrast to nasal pump sprays, pulsating aerosols can deliver significant doses into posterior nasal spaces and paranasal sinuses, providing alternative therapy options before and after sinus surgery. Patients with chronic lung diseases based on clearance dysfunction may also benefit from pulsating aerosols, since these diseases also manifest in the upper airways.

## Introduction

Chronic rhinosinusitis (CRS) is a common chronic disease of the upper airways affecting approximately 10-15% of the US and European population [1,2]. The etiology of CRS is partially unclear, but it is assumed that one or more factors, such as anatomical obstruction, bacterial or fungal colonization of the sinuses or allergies trigger a chronic inflammation of the nasal and paranasal mucosa [3]. The high number of surgical interventions in patients suffering from CRS [4] indicates that medical treatment by oral, systemic or topical drug administration is unsatisfactory and new treatment options are needed. Because of many similarities in anatomy and function between upper and lower airways [5], CRS may also manifest in lung diseases with dysfunctions of the mucociliary apparatus, such as patients with cystic fibrosis (CF) or primary ciliary dyskinesia (PCD) [6]. In addition there is epidemiological evidence for an increased rate of CRS in asthmatics [7] [8].

Topical treatment options for patients with CRS include nasal saline irrigations with a number of different application systems (nasal douches or neti pots) and different saline concentrations (hypertonic vs. isotonic) [9] as well as the topical application of steroids by nasal pump sprays [10,11]. Efficacy is high but in many cases not enough to permanently cure the patient, thus surgical procedures have to be performed. Topical drug delivery to the sinuses via aerosols appears to be an interesting but also challenging alternative, since the paranasal cavities are virtually non-ventilated, poorly perfused, hollow organs protected by the efficient particle filtration function of the nose [12]. In general, research on inhaled topical aerosol treatment of upper airway diseases is desirable but has been “significantly neglected” [13] compared to pulmonary drug investigations.

A scientifically comprehensible approach of aerosol transport to the sinuses is via pressure differences using so-called “pulsating”, ”sonic”, ”acoustic” or “pulsating” aerosols. An aerosol stream superimposed by a vibration or pulsation, i.e. a sound as generated by humming, is called a pulsating aerosol (25 Hz sound used in our study). Similarly, as used for therapy of lung diseases, the aerosol particle can contain a drug formulation, thus enabling an approach for topical administration. Delivery of the drug directly to the site of the disease has the benefit of achieving high local doses and minimizing side effects [14]. For therapy of upper airway diseases nasal pump sprays e.g. containing decongestants are of common use [15,16]. However, as described above, since the sinuses are not ventilated during normal breathing, aerosolized drug delivery to the sinuses is limited.

Early papers on pulsating aerosols suggest that pressure fluctuations enhance the transport of aerosol particles and allow them to penetrate into non-ventilated spaces [17,18]. A more recent study demonstrated significant reduction of exhaled nitric oxide (NO) after administering aerosolized NO synthase inhibitor during humming in healthy volunteers [19]. In previous studies using radioactive ^81m^Kr gas we could demonstrate efficient ventilation of the paranasal sinuses via pressure vibrations while there was no ventilation during normal breathing [20]. In addition sinus deposition of ^99m^Tc-DTPA radiolabeled aerosol in healthy volunteers could be demonstrated, using a commercial pulsating aerosol device. Furthermore, encouraging results from a double-blind placebo controlled trial in CF patients using the PARI SINUS with Dornase-alpha were published recently [21].

Successful aerosolized drug delivery to the sinuses requires small droplets with a mass median aerodynamic diameter (MMAD) of less than 5 µm. Larger droplets, as generated by nasal pump sprays, are filtered by the nose and are not able to reach posterior nasal regions [22]. Moreover, only small particles are able to follow the induced airflow to the sinuses [18].

Previous studies using pulsating aerosols have shown successful sinus delivery in healthy volunteers, but dosimetry data in patients suffering from CRS are not available. The purpose of this study was to assess nasal and sinus aerosol delivery in CRS patients before and after functional endoscopic sinus surgery (FESS) in comparison to healthy volunteers. Although sinonasal lavage is extremely effective after surgery [11], there is currently a high rate of recurrence, therefore additional topical therapy options are also needed after FESS. The study hypothesis was not to prove the clinical efficacy of the pulsating aerosol technique rather than performing a pre-clinical proof of concept dosimetry study. Based on our results studies with clinical end-point evaluation should now be performed.

## Materials and Methods

### Study Participants

11 healthy, non-smoking volunteers participated in this study with mean age of 50+/-12 years (Table 1). In healthy volunteers pulsating aerosols were applied as well as a standard nasal pump spray. In addition 11 patients suffering from chronic rhinosinusitis (CRS) without nasal polyposis (CRSsNP) according to EP3OS criteria [3] participated in the study (mean age 37+/-12 years, Table 1 and paragraph ‘Volunteers’ in File S1). Prior to surgery each patients received a CT scan and severity of CRS was evaluated using the Lund-Mackay score staging (Table 1 and paragraph ‘Volunteers’ in File S1) [23,24]. None of the patients had received FESS in a previous treatment. One patient had septoplasty and conchotomy on both sides eight years before participation in the study. All patients received topical nasal steroids at least for 8 weeks before surgery, postoperatively all patients were treated for another 6 weeks with topical nasal steroids and saline irrigation once daily. In CRS patients pulsating aerosols were applied before and after sinus surgery (FESS). The time between surgery and the second ^99m^Tc-DTPA deposition measurement was 142.7+/-47.7 days (median 145.0 days, range 79.0-264.0 days). On the day of ^99m^Tc -DTPA deposition measurement neither healthy volunteers nor patients did take any medication, such as topical decongestants and/or topical nasal steroid sprays nor irrigations. The study protocol was approved by the Ethical Committee of the Medical School of the Ludwig Maximilian University (Munich, Germany), and written consent was obtained from each subject.

**Table 1:** Anthropometric data and prior-surgery CT based Lund-Mackay-score of the eleven CRS patients and the eleven healthy volunteers participating in the study (NS – non-smokers, S – smokers, XS – ex-smokers, *: p < 0.05 versus healthy, n.d. – not determined).

	Healthy	CRS
Male/Female	9/2	8/3
NS/S/XS	8/0/3	5/6/0
Age, Years	50+/-12	37+/-13*
Height, cm	177+/-10	176+/-10
Weight, kg	77+/-11	81+/-11
Lund-Mackay-score	n.d.	8.2+/-4.0

### Pulsating Aerosol Delivery

A pulsating aerosol was produced using the Vibrent nebulizer prototype (PARI Pharma GmbH, Starnberg, Germany; paragraph ‘Pulsating Aerosol Device and Application’ and Figure S1 in File S1), where a pressure wave of 25Hz was superimposed onto a low velocity (3L/min) aerosol stream. The mass median diameter (MMD) of the aerosol generated by the Vibrent nebulizer was 3.0 µm with a geometric standard deviation of 1.6. For deposition assessment a radiolabeled aerosol was generated using a ^99m^Tc-DTPA solution (Pentacis, Schering, Germany). The nebulizer was inserted into the right nostril, while an exit filter was connected to the left nostril. The aerosol was delivered for 20 seconds, while the subject closed the soft palate. Nebulizer and exit filter were then interchanged and delivery was repeated. Prior to delivery the output rate of the nebulizer was measured. The total nasal deposition rate was assessed from the nebulizer output rate and the exit filter activity.

### Nasal Pump Spray Administration

In healthy volunteers one puff of a nasal pump spray (NS) was administered into each nostril, using a standard 100 µl nasal spray pump from a major manufacturer. Prior to aerosol delivery the output of the pump spray was measured.

### Analysis of Deposition Distribution

**Figure 1:**
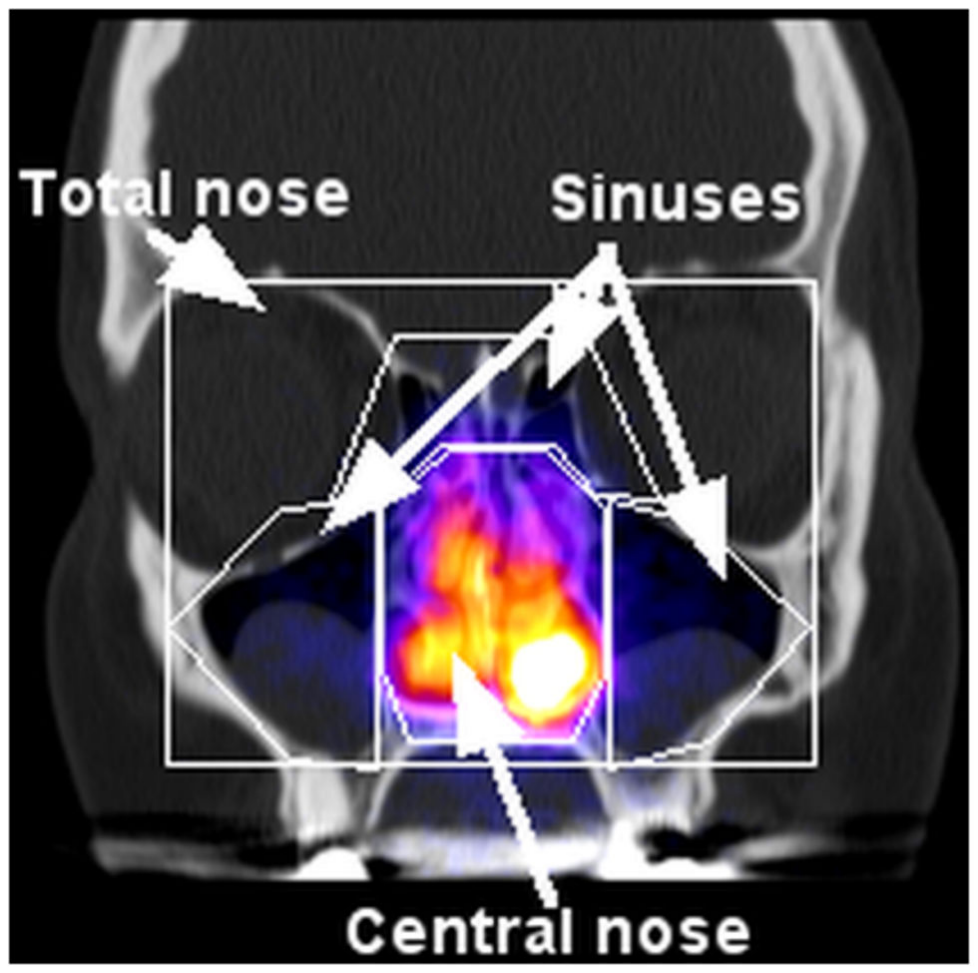
Definition of regions of interest (ROI’s) in anterior gamma camera images. Superposition of the anterior gamma camera image with a representative coronal CT slice of a CRS patient before FESS and definition of the total nasal, central nasal and sinus ROI’s.

**Figure 2:**
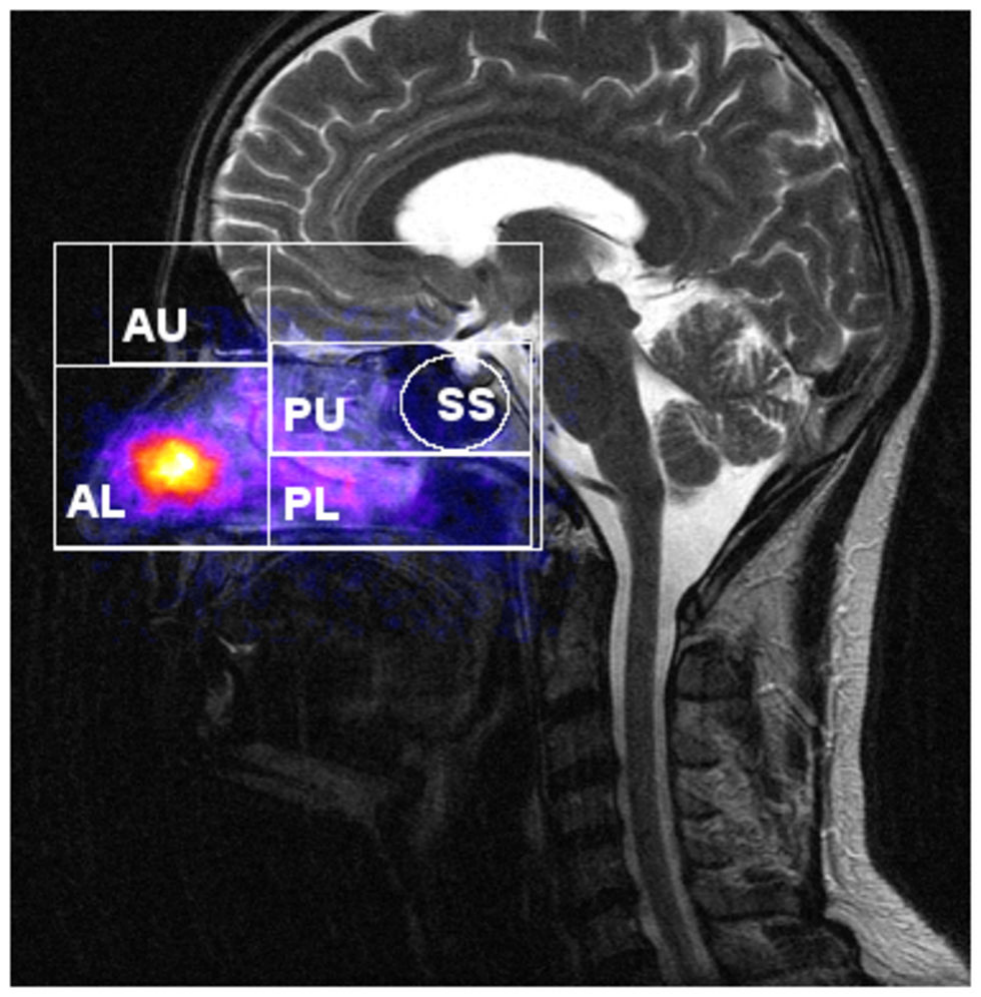
Definition of regions of interest (ROI’s) in lateral gamma camera images. Superposition of the lateral gamma camera image with a representative sagittal CT slice in a healthy volunteer and definition of the different ROI’s: total nasal (TN), anterior upper (AU: frontal sinuses), anterior lower (AL: nostrils, nasal valve and first cm of inferior turbinate), posterior lower (PL: turbinates, nasal floor, hard and soft palate), and posterior upper (PU: upper posterior nasal cavity, middle turbinate, ethmoidal and sphenoidal sinuses), and sphenoidal sinuses (SS).

Nasal and lung deposition were measured directly after inhalation using planar gamma camera imaging (Orbiter, Siemens, Erlangen, Germany). Anterior images were recorded without and with a nasal lead mask (LM, paragraph ‘Gamma Camera Imaging’ and Figure S2 in File S1), allowing visualization of activity in the maxillary and frontal sinuses. Regions of interest (ROI’s) were generated after superposition of the gamma camera image with a representative individual coronal CT or MRI slice of each participant (Figure 1). Similarly, lateral activity distribution was assessed after superposition of the lateral gamma camera image with a representative individual sagittal CT (MRI) slice (Figure 2). The lateral activity was assessed in six different anatomical regions: total nasal (TN), anterior upper (AU), anterior lower (AL), posterior lower (PL), posterior upper (PU) and sphenoidal sinuses (SS). Because anatomy in the lateral images is very complex, the planar projection of the PL and PU compartments (Figure 2) may be a superposition of activity in the nasal cavity, the turbinates and the maxillary, ethmoidal and sphenoidal sinuses. Within PU the sphenoidal sinuses (SS) ROI was defined, which has no overlay with other nasal compartments. Fractional regional deposition was assessed after normalizing to TN, respectively. For estimating the deposited dose in all sinuses relative to total nasal deposition the frontal image results (with and without lead mask shield) were combined with the lateral SS results. Anterior and lateral attenuation correction factors (ACF_A_ and ACF_L_) were determined (paragraph ‘Gamma Camera Imaging’ in File S1).

### Data Analysis

Data were evaluated using Winstat 2009.1 for Excel. All data are presented as mean+/-standard deviation (SD), median, minimum and maximum values. Differences between groups or application modes were assessed by two sided t-test using a significance level of p < 0.05. In addition, differences between application modes in a volunteer group were assessed using a paired t-test when mentioned. Pearson correlation analysis was applied to assess correlation between study variables.

## Results

### Sinus Ventilation

Proper sinus ventilation is a pre-requisite of aerosol transport into the sinuses. As shown in Figure S3 of File S1 and Video S1 significant ventilation of the paranasal sinuses could only be observed when pulsating airflow was applied. There was no Kr-gas penetration into the sinuses during nasal gas passage without vibration.

### Nasal Pump Spray and Pulsating Aerosol Deposition in Healthy Volunteers

**Figure 3:**
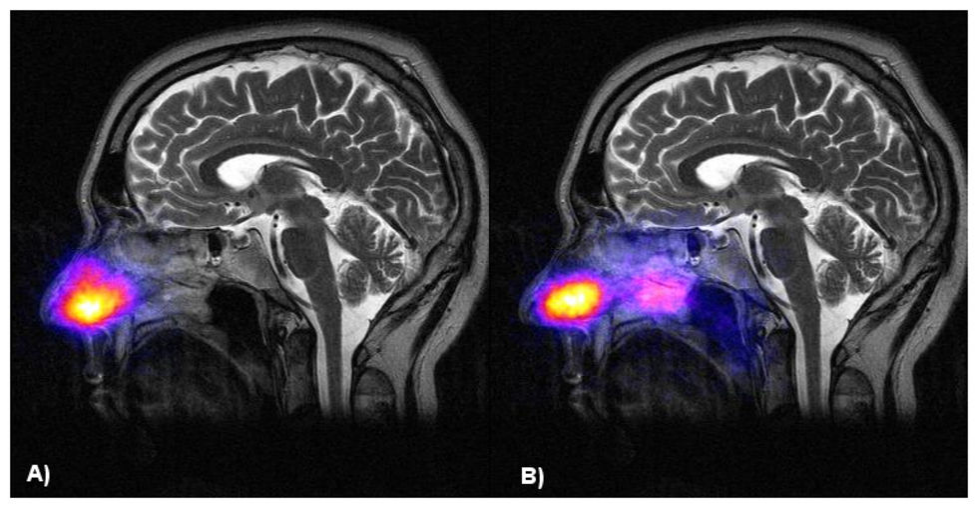
Comparison of deposition distribution of nasal pump spray and pulsating aerosol application. Lateral deposition distribution of nasal pump spray (A) and pulsating aerosol application (B) in a healthy volunteer (superposition of the lateral gamma camera image with an individual representative sagittal MRI slice).

In healthy volunteers ^99m^Tc-DTPA aerosol deposition in the nasal cavity and the paranasal sinuses was studied after nasal pump spray (NS) application and after pulsating aerosol (PA) delivery (Figure 3). The first anterior gamma camera image recorded immediately after aerosol delivery did not show any aerosol deposition in the chest, confirming tight closure of the soft palate during PA delivery. The anterior image with and without nasal lead mask (LM) shielding demonstrated clear deposition of radioactivity in the maxillary sinuses in all volunteers after PA delivery. As shown in Table 2, 61.3+/-8.6% of the administered activity deposited in the total nasal cavity (including sinuses) of the healthy volunteers, while the remaining 40% were expelled from the contralateral nostril and collected on the exit filter. In healthy volunteers 7.1+/-1.7% and 5.7+/-1.9% of the nasally deposited activity could be detected in maxillary sinuses using the anatomy adapted ROI’s without and with LM shielding, respectively. When applying the activity by nasal pumps spray (NS) in healthy volunteers there was 100% deposition in the nasal cavity, no activity penetration into the lungs, and the fraction deposited in the sinuses was below 2% (1.8+/-0.2%) of the nasally deposited activity both without and with nasal LM shielding.

**Table 2:** Total nasal deposition (% nebulizer output) and maxillary sinus deposition (% total nasal deposition) assessed without and with lead mask (LM) shielding after pulsating aerosol application and anterior gamma camera imaging in healthy volunteers and in chronic rhinosinusitis patients (CRS) before and after sinus surgery (FESS).

	Total nasal deposition (% output)	Maxillary sinus deposition (% nasal dose)	Maxillary sinus deposition (LM shield) (% nasal dose)
*Healthy volunteers*			
Mean +/- SD	61.3+/-8.6	7.1+/-1.7	5.7+/-1.9
Median (min, max)	59.3 (47.9, 75.2)	6.2 (5.0, 10.3)	5.8 (3.1, 9.4)
*CRS before FESS*			
Mean +/- SD	56.7+/-13.3	4.0+/-1.7^**^	3.7+/-2.5^*^
Median (min, max)	56.3 (36.1, 77.0)	3.6 (1.8, 7.6)	3.5 (0.8, 8.2)
*CRS after FESS*			
Mean +/- SD	46.7+/-12.7^**,+^	6.1+/-2.2^++^	4.9+/-2.9^++^
Median (min, max)	44.8 (25.1, 65.4)	5.5 (3.1, 10.1)	4.0 (1.2, 8.8)

*= p < 0.05 and **= p < 0.01: compared to healthy volunteers; += p < 0.05 and ++= p < 0.01: CRS before to after FESS.

The lateral image analysis can discriminate deposition distribution as illustrated in Figure 2: anterior upper (AU), anterior lower (AL), posterior lower (PL), posterior upper (PU) and sphenoidal sinuses (SS). Lateral deposition fractions in healthy volunteers after pulsating aerosol (PA) delivery were 65.0+/-10.6%, 19.4+/-9.3%, 11.0+/-4.2% and 2.6+/-1.1% of the total nasal deposition in the AL, PL, PU and SS compartment, respectively (Table 3). Lateral deposition fractions in healthy volunteers after nasal pump spray application were 59.4+/-17.8%, 31.0+/-19.3% (p < 0.05 compared to PA), 5.1+/-2.7% (p < 0.01 compared to PA) and 0.5+/-0.2% (p < 0.01 compared to PA) of the total nasal deposition in AL, PL, PU and SS compartments, respectively. In healthy volunteers the deposited fraction in the frontal sinuses (AU) was below 1% both for PA and NS application.

**Table 3:** Lateral analysis of nasal deposition (mean +/- standard deviation, median, minimum and maximum) in different compartments according to ROI’s as defined in Figure 2 in percent of total lateral nasal deposition (TN): anterior lower (AL) = nostrils and nasal valve; anterior upper (AU) = frontal sinuses; posterior lower (PL) = turbinates, nasal floor, hard and soft palate; posterior upper (PU) = upper posterior nasal cavity, middle turbinate, ethmoidal and sphenoidal sinuses; SS = sphenoidal sinuses.

	**AL % TN**	**PL % TN**	**AU % TN**	**PU % TN**	**SS % TN**
*Healthy-PA*					
Mean +/- SD	65.0+/-10.6	19.4+/-9.3	0.9+/-0.6	11.0+/-4.2	2.6+/-1.1
Median (min, max)	68.3 (40.5, 79.9)	21.0 (5.2, 39.0)	0.9 (0.3, 2.1)	11.4 (5.2, 17.3)	2.9 (1.0, 4.4)
*Healthy-NS*					
Mean +/- SD	59.4+/-17.8	31.0+/-19.3^*^	0.7+/-0.4	5.1+/-2.7^**^	0.5+/-0.3^**^
Median (min, max)	67.4 (36.5, 82.7)	23.1 (5.1, 56.8)	0.5 (0.3, 1.5)	4.3 (1.9, 9.7)	0.5 (0.3, 0.9)
*CRS-PA before FESS*					
Mean +/- SD	63.3+/-8.5	28.3+/-9.9^*^	0.6+/-0.8	5.9+/-4.1^*^	0.8+/-0.6^**^
Median (min, max)	62.4 (49.4, 74.8)	28.6 (14.8, 51.3)	0.4 (0.1, 2.8)	4.3 (1.5, 14.9)	0.5 (0.2, 1.9)
*CRS-PA after FESS*					
Mean +/- SD	59.6+/-13.9	29.8+/-12.6^*^	0.6+/-0.6	9.0+/-4.0^+^	2.1+/-1.8^++^
Median (min, max)	55.5 (40.2, 83.5)	30.1 (12.9, 52.0)	0.4 (0.1, 2.3)	8.4 (2.9, 16.5)	1.3 (0.7, 6.0)

Pulsating aerosols (PA) were applied in healthy volunteers and in chronic rhinosinusitis (CRS) patients before and after sinus surgery (FESS). In addition nasal pump sprays (NS) were applied in healthy volunteers. * p < 0.05 compared to Healthy-PA, ** p < 0.01 compared to Healthy-PA, + p < 0.05 CRS before versus after surgery, ++ p < 0.01 CRS before versus after surgery.

### Pulsating Aerosol Deposition in CRS Patients Before and After Sinus Surgery (FESS)

**Figure 4:**
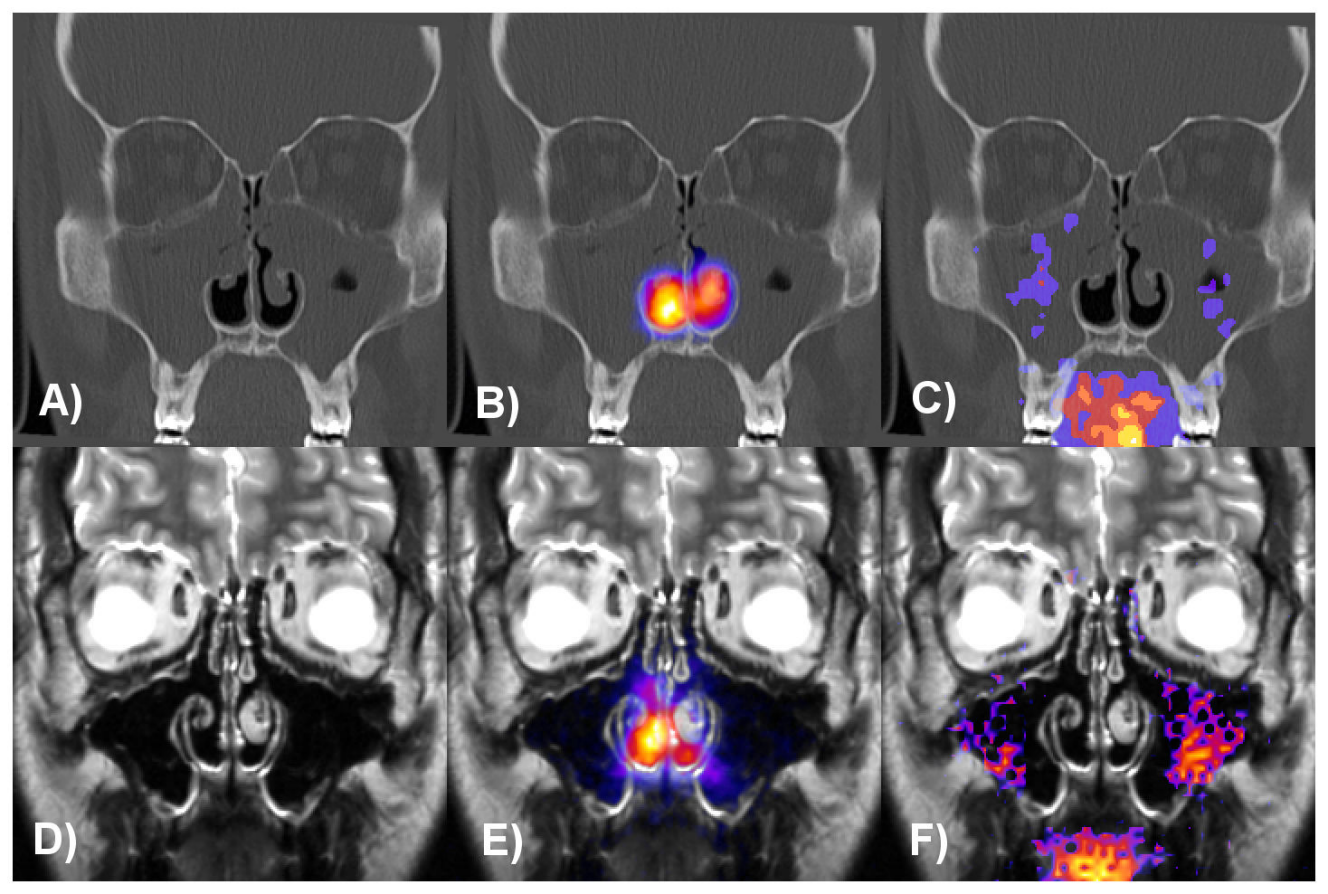
Coronal CT and MRI slices of a CRS patient before and 166 days after sinus surgery. Superposition of the anterior gamma camera images with the CT slice before FESS (A–C) without (B) and with (C) central nasal lead shield mask and with the MRI slice after FESS (D–F) without (E) and with (F) lead shield. Lund-Mackay score was 15 and maxillary sinus deposition was 1.3% (C) before and 7.9% (F) after surgery. The patient had septoplasty and conchotomy on both sides eight years before participation in the study.

**Figure 5:**
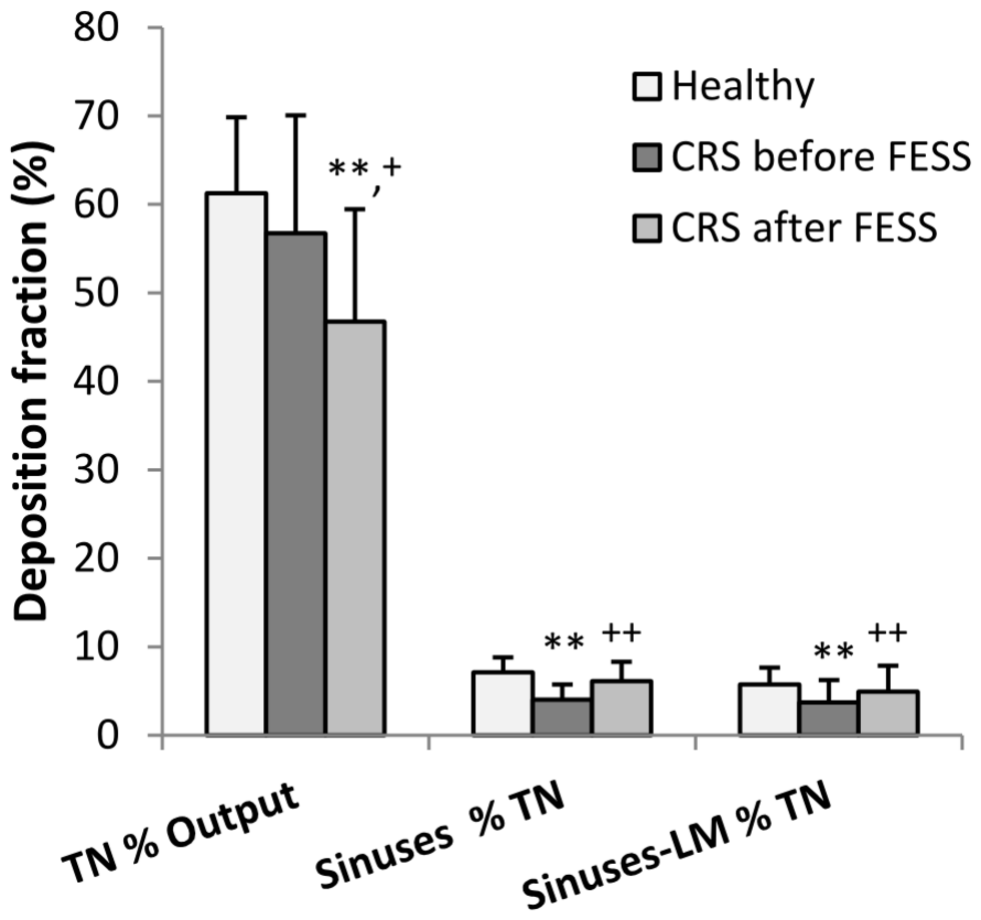
Analysis of deposition fractions from anterior gamma camera imaging. Anterior deposition fractions in total nose (TN % nebulizer output) and in maxillary sinuses without and with lead mask (LM) shielding of the central nasal cavity (% total nasal deposition, TN) in healthy volunteers and in CRS patients before and after sinus surgery (FESS) after pulsating aerosol delivery. **: p < 0.01 compared to healthy; +: p < 0.05 and ++: p < 0.01 before versus after FESS in CRS patients.

For one CRS patient Figure 4 shows coronal CT slices (A-C) before and MRI slices (D–F) 166 days after sinus surgery. In addition superposition of the CT slices with anterior gamma camera images before FESS without (B) and with (C) central nasal lead shield mask as well as MRI slices (after FESS) without (E) and with (F) lead shield are shown. Before functional endoscopic sinus surgery (FESS), 56.7+/-13.3% of the administered activity deposited in the total nasal cavity of the CRS patients and 4.0+/-1.7% and 3.7+/-2.5% of the nasally deposited activity were detected in the maxillary sinuses without and with LM shielding, respectively (p < 0.01 compared to healthy, Table 2 and Figure 5). Maxillary sinus deposition inversely correlated with the Lund-Mackay score (coefficient of correlation, cc = -0.53, p < 0.05). 143+/-48 days after FESS, 46.7+/-12.7% (p < 0.01 comp. to healthy-PA; p < 0.05 compared to CRS prior to FESS, paired t-test) of the administered activity deposited in the total nasal cavity of the CRS patients and 6.1+/-2.2% and 4.9+/-2.9% of the nasally deposited activity were detected in the sinuses without and with LM shielding, respectively (p < 0.01 compared to CRS before FESS, paired t-test; no significant difference to healthy volunteers).

**Figure 6:**
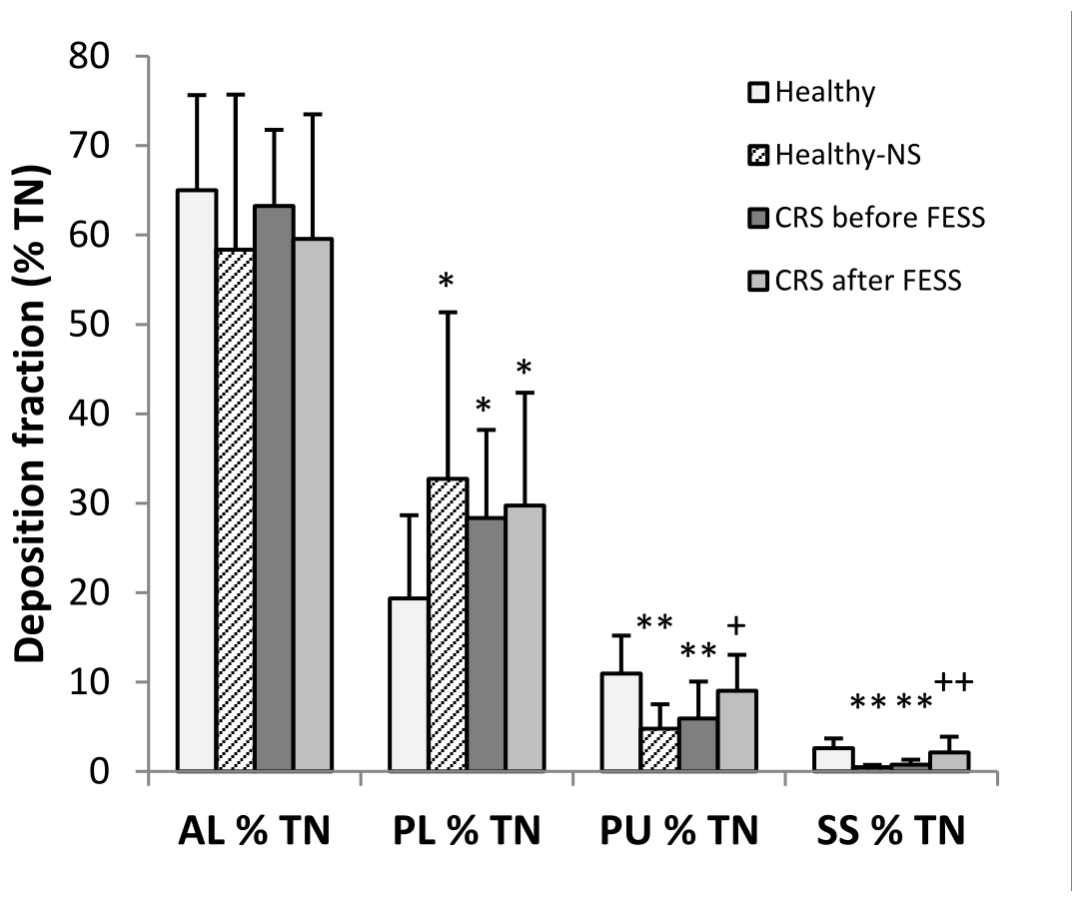
Analysis of deposition fractions from lateral gamma camera imaging. Lateral nasal deposition fractions (mean +/- standard deviation) in different compartments according to ROI’s as defined in Figure 2 in percent of total lateral nasal deposition (% TN): anterior lower (AL) = nostrils and nasal valve; posterior lower (PL) = turbinates, nasal floor, hard and soft palate; posterior upper (PU) = upper posterior nasal cavity, middle turbinate, ethmoidal and sphenoid sinuses; sphenoidal sinuses (SS). Pulsating aerosols were applied in healthy volunteers and in CRS patients before and after sinus surgery (FESS). In addition nasal pump sprays (NS) were applied in healthy volunteers. The AU analysis (frontal sinuses) was not included because deposition was below 1% in all volunteers and application modes studied. *: p < 0.05 and **: p < 0.01 compared to healthy volunteers; +: p < 0.05 ++: p < 0.01 before versus after FESS in CRS patients.

Before FESS, CRS patients showed 63.4+/-8.4%, 27.8+/-9.8%, 6.6+/-6.0% and 0.8+/-0.6% of the total nasal deposition in the AL, PL, PU and SS compartment after lateral imaging, respectively (Table 3 and Figure 6). 143+/-48 days after FESS, CRS patients showed 59.6+/-13.9%, 29.8+/-12.6%, 9.0+/-4.0% (p < 0.05 compared to CRS before FESS, paired t-test) and 2.1+/-1.8% (p < 0.01 compared to CRS before FESS, paired t-test) of the total nasal deposition in AL, PL, PU and SS compartments after lateral imaging, respectively. Activity deposited in AU (frontal sinuses) was below 1% before and after FESS, which is considered being non-significant.

After pulsating aerosol delivery total maxillary and sphenoidal sinus deposition (estimated from anterior and lateral imaging) was 9.7+/-2.0% in healthy volunteers, and 4.8+/-2.2% (p < 0.01 compared to healthy-PA) and 8.2+/-3.8% (p < 0.01 compared to CRS before FESS) in CRS patients before and after sinus surgery (FESS), respectively.

Among all volunteers and application modes there was an inverse correlation between total deposited nasal dose and sinus deposition (coefficient of correlation, cc = -0.55, p < 0.01). A high correlation was observed between maxillary sinus deposition assessed without and with LM shielding (cc = 0.86, p < 0.01). Deposition in the anterior and posterior lower compartments (AL and PL) were inversely correlated (cc = -0.92, p < 0.01). Fractional deposition in the lateral PU- and SS-compartments showed a negative correlation with total nasal deposition (cc = -0.43, p < 0.01, and cc = -0.42, p < 0.01, respectively), but a significant positive correlation with maxillary sinus deposition assessed by anterior imaging (cc = 0.65, p < 0.01, and cc = 0.75, p < 0.01, respectively), both without and with LM shielding.

### Attenuation Correction Factors

**Figure 7:**
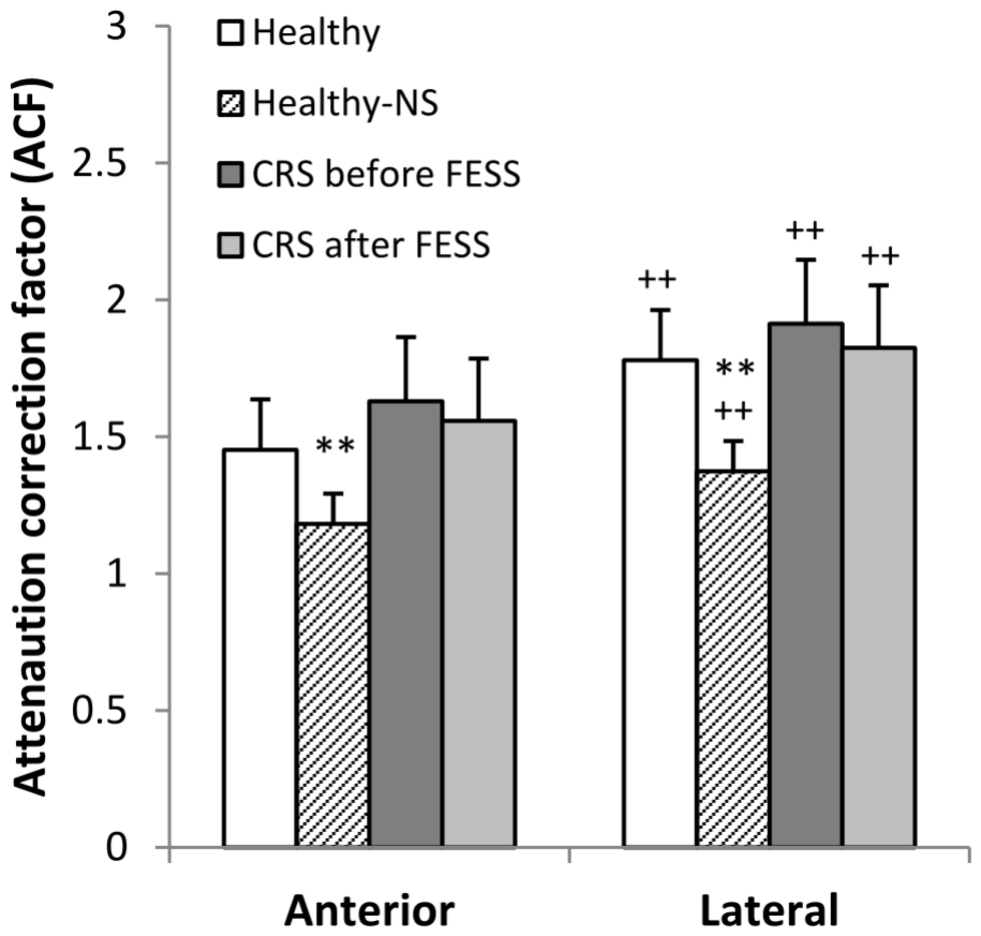
Analysis of attenuation correction factors after anterior and lateral gamma camera imaging. Anterior and lateral attenuation correction factors (ACF) after pulsating aerosol (PA) delivery in healthy volunteers and in CRS patients before and after sinus surgery (FESS) as well as in healthy volunteers after nasal spray application (Healthy-NS). **: p < 0.01 compared to Healthy-PA, ++: p < 0.01 lateral versus anterior imaging.

As shown in Table 4 and Figure 7, lateral attenuation correction factors, ACF_L_ were always higher compared to anterior ACF_A_, in all volunteers studied for all application modes (p < 0.01). Using pulsating aerosol application in healthy volunteers ACF_A_ was 1.45+/-0.18 and ACF_L_ was 1.78+/-0.32, and ACF_A_ and ACF_L_ were not significantly different in CRS patients before and after FESS compared to healthy volunteers after pulsating aerosol delivery. However, ACF_A_ was 1.18+/-0.11 and ACF_L_ was 1.37+/-0.19 in healthy volunteers after nasal spray application (p < 0.01 compared to PA application). In all volunteers ACF_A_ and ACF_L_ were highly correlated (cc =0.9, p < 0.01). ACF_L_ showed a significant correlation with fractional deposition in the PU-compartment (cc = 0.42, p < 0.01).

**Table 4:** Anterior and lateral attenuation correction factors (ACF) after pulsating aerosol (PA) delivery in healthy volunteers and in CRS patients before and after sinus surgery (FESS) as well as in healthy volunteers after nasal spray application (Healthy-NS).

	**Anterior ACF_A_**	**Lateral ACF_L_**
*Healthy-PA*		
Mean +/- SD	1.45+/-0.18	1.77+/-0.31^++^
Median (min, max)	1.54 (1.07, 1.64)	1.82 (1.25, 2.21)
*Healthy-NS*		
Mean +/- SD	1.18+/-0.11^**^	1.37+/-0.19^**,++^
Median (min, max)	1.17 (1.06, 1.36)	1.35 (1.12, 1.75)
*CRS-PA before FESS*		
Mean +/- SD	1.64+/-0.23	1.91+/-0.28^++^
Median (min, max)	1.53 (1.30, 2.14)	1.83 (1.51, 2.36)
*CRS-PA after FESS*		
Mean +/- SD	1.56+/-0.23	1.82+/-0.27^++^
Median (min, max)	1.57 (1.22, 2.06)	1.82 (1.44, 2.29)

** p < 0.01 compared to Healthy-PA, ++ p < 0.01 lateral versus anterior imaging.

## Discussion

### Sinus Ventilation

During normal breathing the typical gas exchange time constant between the nasal cavity and the maxillary sinuses was 8-10 min, as measured in healthy volunteers by serial Xe-gas-enhanced CT-imaging [25]. This could be confirmed in our studies using ^81m^Kr gas ventilation gamma camera imaging [20], where no Kr-gas penetration occurred into the maxillary sinuses during a 20 sec application without vibration. During pulsation, there was Kr-gas penetration into the maxillary, ethmoidal and sphenoidal sinuses in healthy volunteers, confirming effective ventilation of the sinuses, a major pre-requisite for pulsating aerosol delivery (see Figure S3 in File S1 and Video S1). However, in most healthy volunteers no significant Kr-gas penetration could be detected in the frontal sinuses, which correlates with non-significant aerosol deposition.

### Nasal and Sinus Aerosol Deposition

Similarly, as shown in a previous study using the PARI Sinus [26], the pulsating aerosol technique does not deposit the whole emitted dose in the nasal cavity and between 30 and 40% of the emitted dose was expelled through the exit nostril and collected on the exit filter. This is different from the application of nasal pumps sprays, where 100% of the emitted dose are deposited in the nose. 60% nasal deposition efficiency is in agreement with experimental data of aerosol deposition in the nasal cavity during nose breathing [22] and confirms the superior properties of the generated fine aerosol (3.0 µm MMD of the Vibrent prototype versus 60 µm of conventional pump spray systems), which can penetrate into the posterior regions of the nasal cavity, where the entrances to the sinuses (ostia) are located. This deeper penetration is confirmed in our study in healthy volunteers when analyzing the lateral deposition distribution. As shown in Figure 3A, the major activity deposition was in the nostrils and the nasal valve after nasal spray (NS) application and no significant activity could be detected in the sphenoidal sinuses (SS). In contrast pulsating aerosol (PA) delivery deposited significantly higher fractions in the posterior upper region (PU, includes ethmoidal and sphenoidal space) of the nose (Figure 3B and Table 3). Higher PU and SS deposition of PA compared to NS application could also be confirmed in CRS patients before and after sinus surgery.

Although at higher range there is no significant difference in total nasal deposition of PA in CRS patients prior to sinus surgery (FESS) compared to healthy volunteers (56.7+/-13.3% in CRS before FESS compared to 61.3+/-8.6% in healthy, Table 2). This was an unexpected finding, as one would expect higher nasal deposition in CRS patients due to the mostly obstructed nasal passages before FESS, therefore generating higher resistance to an aerosol stream, possibly causing higher deposition by impaction. Therefore the nose seems to be an equally effective particle filter in healthy subjects as in CRS patients. Nevertheless, a surprisingly high fraction of the administered aerosol could penetrate into the maxillary sinus cavities of CRS patients prior to FESS (4.8+/-2.2%), where pre-surgery maxillary sinus deposition decreased with increasing severity of CRS, as assessed by the inverse correlation with the Lund-Mackay score. In addition since all CRS patients in our study were without nasal polyps (CRSsNP) pulsating aerosol sinus deposition may further decrease in more severe patients with nasal polyps (CRSwNP) with higher Lund-Mackay scores. In summary, topical pulsating aerosol therapy might be a viable option prior to sinus surgery, but needs confirmation in future clinical studies.

At least two months after FESS (143+/-48 days) the deposition efficiencies are changing in the CRS patients and total nasal deposition decreased significantly (46.7+/-12.7% after FESS versus 56.7+/-13.3% before FESS, p < 0.01) using similar operation parameters of the device. This decrease may be related to the resolution of the obstruction and the enlargement of the nasal passages during surgery. Simultaneously there was a significant increase in maxillary and sphenoidal sinus deposition after FESS (8.2+/-3.8%) and the data reach a level comparable to healthy volunteer’s (9.7+/-2.0%). However, the expected further increase in sinus deposition due to enlargement of the ostia beyond the value measured in healthy volunteers could not be confirmed. The data of the healthy volunteers seem to indicate an upper threshold for sinus delivery of pulsating aerosols using the present device configuration. Nevertheless, since the physical boundary conditions changed after sinus surgery, the system may be de-tuned after FESS [27]. Therefore, the applied protocol of the pulsating aerosol (frequency and pressure amplitude) may be no more optimal for maximum ventilation and aerosol penetration to the sinuses in CRS patients after sinus surgery and requires further optimization.

Lateral distribution analysis allowed discriminating deposited fractions in the anterior and posterior, as well as in the upper and lower nasal cavity, respectively. Because of the complex anatomy, lateral gamma camera images may show a superposition of different anatomical objects. I.e. the PL compartment is a planar projection of activity in maxillary sinuses, the inferior turbinate and the nasal floor. The PU compartment relies on the upper turbinate, the upper nasal cavity and the ethmoidal and sphenoidal sinuses. However, as shown in Figure 2, the sphenoidal sinuses could be extracted from the PU compartment as an additional sinus-ROI without interference from other tissues. Therefore the total assessed sinus deposition was obtained from anterior gamma camera imaging (maxillary sinuses) and the lateral sphenoidal sinus efficiency. Neither in healthy volunteers nor in CRS patients before and after FESS there was significant aerosol deposition in the frontal sinuses. This correlates with limited Kr-gas ventilation of frontal sinuses in healthy volunteers and allows concluding that the PA technique with parameters used in this study cannot deliver significant amounts of aerosol into this region.

The lateral images showed that the major fraction of activity was deposited at the nostrils, the nasal valve and the anterior inferior turbinates (AL, 65% in healthy volunteers and 59% and 62% in CRS patients before and after FESS, respectively). However, a significant fraction (35-40%) could penetrate into the deeper posterior nasal cavity (PL and PU) where the ostia provide access into the sinuses. For proper interpretation it has further to be considered that the lateral PL compartment in part is fed by mucociliary transport from the AL compartment, making it also a transit compartment [15].

### Aerosol Deposition Distribution and ACF

Using the masking technique with a nasal LM shield, deposition of pulsating aerosols in the maxillary sinuses could be clearly confirmed. While qualitative proof of (maxillary) sinus deposition is straight forward, the quantitative determination is not as evident from planar gamma camera imaging. Compton scattering and low resolution of the camera might lead to detection of ostensive activity in the sinuses. For example, for nasal sprays 1.8% sinus deposition were found although it is known that nasal sprays do not penetrate the sinuses. Hence, 1.8% sinus deposition may indicate a lower resolution threshold due to gamma ray scattering. In addition, gamma radiation of the deposited activity is attenuated by the surrounding bone and tissue making recovery calculations difficult. Our study shows significant influences of anterior versus lateral imaging on gamma ray attenuation together with the mode of aerosol application (PA versus NS). In all volunteers studied and for all application modes lateral ACF_L_’s were always higher compared to anterior ACF_A_’s (Table 4 and Figure 7), and lateral and anterior ACF’s were highly correlated. More tissue and bone mass is located between the deposited activity and the gamma camera head in lateral compared to anterior view. Lateral ACF’s might in part be influenced by loss of activity due to mucociliary clearance into the oropharynx and subsequent swallowing. But this cannot be a major effect as not more than three minutes elapse between anterior and lateral image recording, where we may have only minute clearance from the nasal cavity [15].

In addition, since the ACF’s (both anterior and lateral) are significantly lower for nasal spray (NS, ACF_L_ ≈ 1.4) compared to pulsating aerosol (PA, ACF_L_ ≈ 1.8) application (Table 4), NS deposition pattern are more anterior and the major fractions have deposited at the nostrils and the nasal valve. This is in agreement with local ACF measurements in nine volunteers by Skretting et al. [28], who got lateral ACF_L_’s of about 1.2 and 2.3 in the anterior and posterior nasal cavity, respectively. The overall ACF’s in our study between 1.2–2 are in agreement with their experimental data [28]. Therefore, higher ACF’s of the pulsating aerosol further confirm penetration into deeper posterior nasal spaces, where access to the ostia and the sinuses is provided.

### Clinical Application

The protocol of application of the pulsating aerosol implies the closure of the soft palate, therefore requiring patient co-operation and compliance. Our previous studies have shown that PA delivery into the sinuses can even be achieved during nasal breathing, although at reduced rate, but still significant to perform aerosol therapy [29]. In these studies lung deposition was below 15% of the emitted dose and there was still activity on the exit filter, showing that a fraction of the aerosol could be exhaled. However, as known from inhalation therapy in various lung diseases, it can be concluded that those patients with more severe conditions (such as CF or severe asthmatic patients) may show the highest compliance in following a suggested treatment protocol (e.g. closing the soft palate) to take the most therapeutic benefit [30,31]. In addition, proper use of any inhalation device may be warranted by repeated training of the patients [32].

Dose calculations have shown that a two minute administration of the pulsating aerosol device can deposit doses in the nasal cavity being comparable to two puffs (200 µl) of a nasal pump spray [33], although the nasal spray do not deliver significant doses to the sinuses. However, based on total nasal and sinus deposition assessed in this study, 15-20 mg of the delivery dose can deposit in the sinuses during a one-min treatment. Therefore, further clinical studies are needed to prove clinical end points of the pulsating aerosol technique and first promising results are already available [21]. In addition as shown in a case study (paragraph ‘Case Study: Two Months Topical Steroid Aerosol Therapy Using Pulsating Aerosols’ in File S1) three patients with CRS used the pulsating aerosol technique to deliver once daily a steroid to the nasal cavities as an alternative to FESS. All nasal and sinus obstructions had disappeared after two months of therapy, as confirmed by fiber optic rhinoscopy and MRI (Figure S4 in File S1). Thus, sinus surgery could be avoided. However, long-term evaluation of these therapies is required.

In summary, the pulsating aerosol drug delivery technology may provide a new topical therapy option for CRS patients. The high rate of surgery in CRS might indicate, that the currently available topical therapies, such as nasal pump sprays, nasal drops or irrigation have limited efficiency [10,11], at least before surgery. Recent studies have shown that biofilms form additional barriers in treatment of CRS, and these may require new drug formulations and therapy protocols [34,35]. Most drug formulations being currently used for topical therapies, such as saline, antibiotics, antifungals and steroids can similarly be used with the pulsating aerosol device. The performance in patients with nasal polyps (CRSwNP) has to be assessed in future studies since all patients in our study were without nasal polyps (CRSsNP). In addition, topical therapies are also needed after surgery and saline irrigation has demonstrated high efficiency in clinical studies [11]. However, pulsating aerosols may similarly be applicable for a post-surgery therapy because of controlled dosing, limited wasting of drug and easy handling. Studies evaluating clinical end-points (i.e. improvement of symptoms, quality of live, prevention of surgery, and evaluation of after surgery therapy) have to be conducted to confirm whether the here documented sinus aerosol delivery may be of clinical and therapeutic relevance. The case study provided in the supplemental file may suggest for such potency.

## Supporting Information

File S1 **Additional information in four paragraphs on Volunteers, Gamma Camera Imaging, ^81m^Kr-Gas Sinus Ventilation Imaging and Case Study: Two Months Topical Steroid Aerosol Therapy Using Pulsating Aerosols, including supporting Figures.** Figure S1: Prototype of the Vibrent® pulsating aerosol device consisting of a pulsating membrane nebulizer with nostril adaptor, flow and 25 Hz pulsation generator and the nasal resistance. Figure S2: Lead mask for shielding the activity in the central nasal cavity during anterior imaging in front of the gamma camera. The lead mask consists of 2 mm lead causing an attenuation of the gamma rays by about a factor of ≈ 900. Figure S3: Gamma camera imaging of ^81m^Kr-gas ventilation of the nasal cavity and the sinuses. Anterior (A and B) and lateral (C and D) imaging of 81mKr-gas ventilation of the nasal cavity and the sinuses without (A and C) and with (B and D) the vibration airflow technology (anterior and lateral gamma camera images superimposed onto coronal and sagittal MRI slices of the volunteer). Figure S4: MRI slices of a patient before and after topical steroid treatment using a pulsating aerosol device. Coronal MRI (T2 weighted) slices of a patient before (left) and after (right) a two months once daily treatment with steroids (Pulmicort respules, 1 mg/2 mL) using the PARI Sinus pulsating aerosol device.
(PDF)

Video S1 **Dynamic gamma camera imaging of ^81m^Kr-gas nasal and sinus ventilation without and with pulsating gas delivery.**
(MOV)
