# Supplementary material for: Topical Drug Delivery in Chronic Rhinosinusitis Patients before and after Sinus Surgery Using Pulsating Aerosols
Source: PLoS One. 2013 Sep 11;8(9):e74991. doi: 10.1371/journal.pone.0074991 (PMC3770586; doi:10.1371/journal.pone.0074991)
Supplement: File S1 — Additional information in four paragraphs on Volunteers, Gamma Camera Imaging, 81mKr-Gas Sinus Ventilation Imaging and Case Study: Two Months Topical Steroid Aerosol Therapy Using Pulsating Aerosols, including supporting Figures. Figure S1: Prototype of the Vibrent® pulsating aerosol device consisting of a pulsating membrane nebulizer with nostril adaptor, flow and 25 Hz pulsation generator and the nasal resistance. Figure S2: Lead mask for shielding the activity in the central nasal cavity during anterior imaging in front of the gamma camera. The lead mask consists of 2 mm lead causing an attenuation of the gamma rays by about a factor of ≈ 900. Figure S3: Gamma camera imaging of 81mKr-gas ventilation of the nasal cavity and the sinuses. Anterior (A and B) and lateral (C and D) imaging of 81mKr-gas ventilation of the nasal cavity and the sinuses without (A and C) and with (B and D) the vibration airflow technology (anterior and lateral gamma camera images superimposed onto coronal and sagittal MRI slices of the volunteer). Figure S4: MRI slices of a patient before and after topical steroid treatment using a pulsating aerosol device. Coronal MRI (T2 weighted) slices of a patient before (left) and after (right) a two months once daily treatment with steroids (Pulmicort respules, 1 mg/2 mL) using the PARI Sinus pulsating aerosol device. (PDF) [file pone.0074991.s001.pdf]

SUPPORTING INFORMATION

Topical drug delivery in chronic rhinosinusitis patients before and after sinus surgery using pulsating aerosols

Volunteers

Eleven healthy, non-smoking volunteers participated in this study with mean age of 50+/-12 years. None of the healthy volunteers was an active smoker. The healthy volunteers had normal lung function and no history of allergic diseases. Normal nasal anatomy was confirmed prior to the study by MRI of the head and fiber optic rhinoscopy. In healthy volunteers pulsating aerosols were applied as well as a standard nasal pump spray with at least two weeks in between administrations.

In addition 11 patients suffering from chronic rhinosinusitis (CRS) without nasal polyposis (CRSsNP) participated in the study (37+/-12 years of age, five non-smokers and six smokers). The anthropometric data of the volunteers are shown in Table 1. Among 20 CRS patients initially recruited 19 agreed to participate in the study. Three of the 19 patients underwent a two months topical steroid therapy (see case study below) using Pulmicort Respules (1 mg/2 ml, once daily) delivered via the PARI Sinus pulsating aerosol device (PARI GmbH, Starnberg, Germany). From the remaining 16 patients reliable data could be derived for 11 patients, each before and after surgery, and analyzed in detail. All 11 patients fulfilled the EP3OS criteria for CRS without nasal polyposis (CRSsNP) (Fokkens, W., et al. (2007). The European Position Paper on Rhinosinusitis and Nasal Polyps (EP3OS) group. Rhinology 45(Suppl. 20), 1-137). Patients were selected from the university outpatient clinic of the Department of Otorhinolaryngology, Head and Neck Surgery of Ludwig Maximilian University in Munich. None of the patients had received FESS in a previous treatment. One of the eleven patients had septoplasty and conchotomy on both sides about 8 years before participation in the study. All patients had received topic nasal corticosteroids and performed nasal irrigation for at least 8 weeks with no improvement of sinunasal symptoms, thus FESS was indicated.

Prior to surgery each patients received a CT scan and severity of CRS was evaluated using the Lund-Mackay score staging (Lund VJ, Mackay IS (1993) Staging in rhinosinusitis. Rhinology 31:183-184; Zinreich SJ (2004) Imaging for staging of rhinosinusitis. Ann Oto Rhinol Laryn 113 (5):19-23). This scale grades the right and left sides independently, looking at the maxillary, anterior ethmoidal, posterior ethmoidal, sphenoidal, and frontal sinuses, as well as the ostiomeatal complex. Each sinus is scored a 0 (no abnormality), 1 (partial opacification), or 2 (total opacification), while the ostiomeatal complex is scored either a 0 or 2 (for presence or absence of disease). All scores are summed and can therefore range from 0 - 24. The pre-surgery Lund-Mackay score in the eleven CRS patients was 8.2+/-4.0 (7.0, 4.0, 16.0; median, min, max). Six of the eleven patients in the FESS group and all patients on two months topical steroid therapy received an MRI scan at the time of the second post-therapy radio-aerosol deposition study.

Pulsating Aerosol Device and Application

Pulsating aerosol delivery system

A pulsating aerosol was produced using the Vibrent® nebulizer prototype (PARI Pharma GmbH, Starnberg, Germany) generating a fine aerosol mist of an aqueous liquid via a perforated pulsating membrane (Figure S1; Knoch, M. and Keller, M. (2005). The customised electronic nebuliser: a new category of liquid aerosol drug delivery systems. Expert Opin. Drug Deliv. 2(2), 377-390). In the Vibrent, a pressure wave of 25 Hz frequency was superimposed on a low velocity (3 L/min) aerosol stream. The mass median diameter (MMD) of the aerosol generated by the Vibrent nebulizer was 3.0 µm with a geometric standard deviation of 1.6. The droplet size distribution of the nebulizer was measured using the Laser Diffraction Technique and no significant difference in droplet size was detected between saline and the ^{99m}Tc-DTPA solution used in our study. The rate of mass output was 0.3 mL/min. An aerosol was generated using a solution containing ^{99m}Tc-DTPA (Pentacis, Schering, Germany). The volunteers did not receive any treatment before application, for example decongesting nasal spray; therefore the nasal cycle was not eliminated.

For nasal aerosol delivery the nebulizer was inserted into the right nostril, while a filter including a flow resistor was connected to the left nostril (PALL BB50 filter, Pall Corporation, New York, USA). The aerosol was delivered for 20 seconds, while the subject closed the soft palate. This generates an unidirectional aerosol flow through the nasal cavity (one nostril in, the other nostril out) without aerosol penetration into the oropharyngeal cavity or the lung. Nebulizer and output filter were then interchanged between left and right nostril and the aerosol was delivered for an additional 20 seconds. Prior to aerosol delivery the output from the nebulizer was measured by collecting all particles on a PALL BB50 filter. From the nebulizer output and the activity deposited on the output filter the total nasal deposition was assessed.

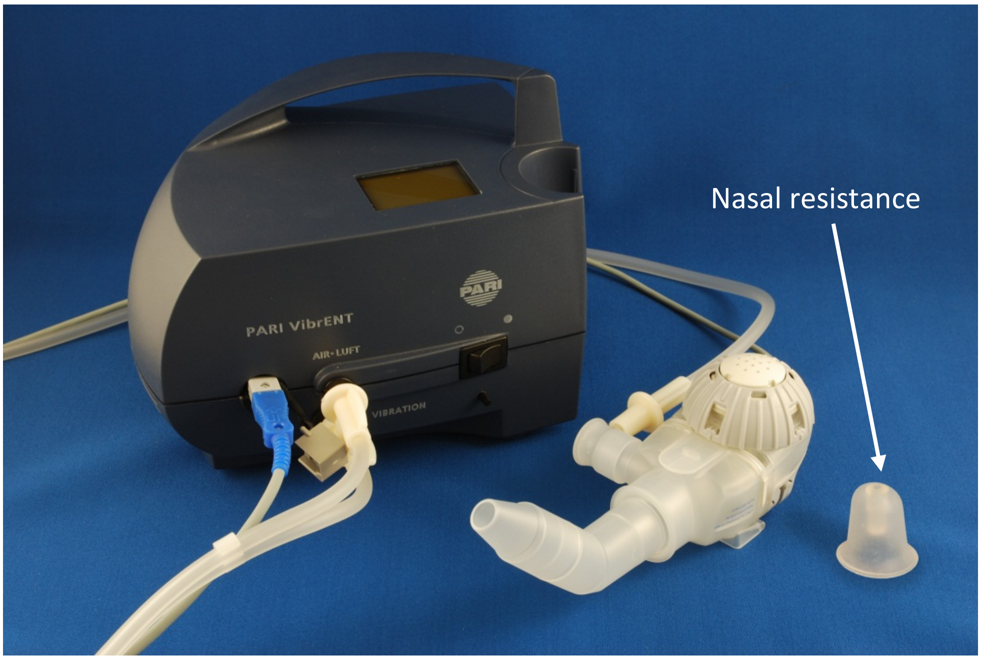

Figure S1: **Prototype of the Vibrent® pulsating aerosol device consisting of a pulsating membrane nebulizer with nostril adaptor, flow and 25 Hz pulsation generator and the nasal resistance.**

Gamma Camera Imaging

Analysis of Deposition Distribution

Nasal and lung deposition were measured using planar gamma camera imaging (Orbiter, Siemens, Erlangen, Germany) in combination with a low energy collimator. Anterior and lateral gamma camera images (60 sec each) were recorded directly after inhalation. The subjects sat in front of the gamma camera head, which was positioned in the upright format, allowing a simultaneous imaging of the nose and the upper lung. Anterior images were recorded without and with a nasal lead mask (LM, Figure S2). Masking the centrally deposited activity allowed clear visualization of the deposited activity in the maxillary, frontal and ethmoidal sinuses (Figure 4, c and f).

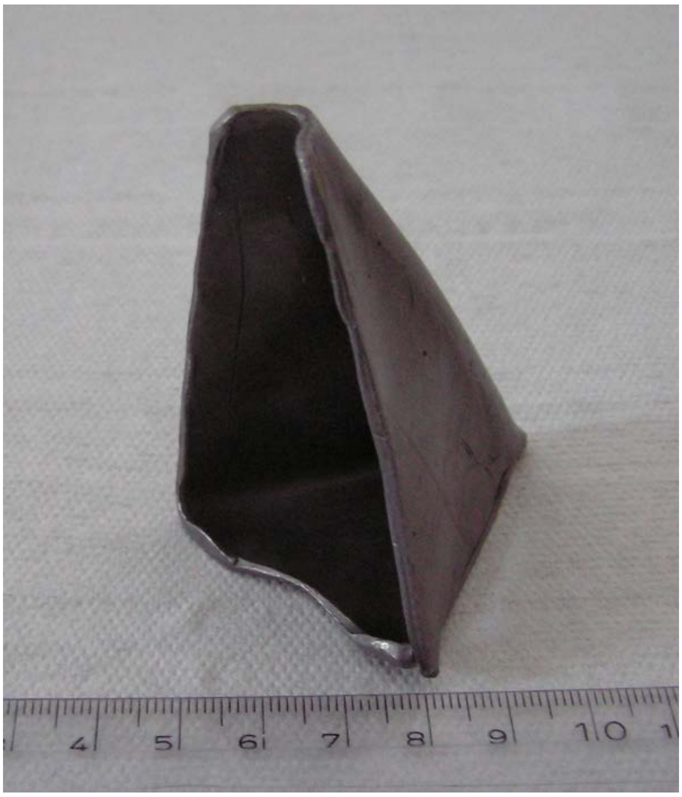

Figure S2: **Lead mask for shielding the activity in the central nasal cavity during anterior imaging in front of the gamma camera.** The lead mask consists of 2 mm lead causing an attenuation of the gamma rays by about a factor of ≈ 900.

Count rates in selected regions of interest (ROI's) were analyzed using the ImageJ software package. ROI's were generated after superposition of the gamma camera image with a representative slice of the coronal CT stack (MRI stack in healthy volunteers), as shown in Figure 1. Regional central nasal and sinus deposition efficiencies were assessed after normalizing to the total nasal activity. Similarly, as shown in Figure 2, lateral activity distribution was assessed after superposition of the lateral gamma camera image with an individual representative slice of the sagittal CT (MRI) stack. In the lateral distribution analysis activity was assessed in six different anatomical areas of the nose: total nasal (TN) = total nasal (excluding nasopharynx); anterior lower (AL) = nostrils, nasal valve and first cm of inferior turbinate; anterior upper (AU) = frontal sinuses; posterior lower (PL) = turbinates, nasal floor, hard and soft palate, posterior upper (PU) = upper nasal cavity and turbinates, ethmoidal and sphenoidal sinuses, and sphenoidal sinuses (SS). Fractional regional deposition distribution was assessed after normalizing AL, AU, PL, PU and SS to total nasal activity (TN), respectively.

Assessment of Attenuation Correction Factors

The deposited dose (DD) was measured based on device output and exit filter evaluation. Based on the deposited dose, anterior and lateral gamma camera count rates (CR_A, CR_L) were used to evaluate anterior and lateral gamma ray attenuation correction factors (ACF_A and ACF_L, respectively) according to:

$$ACF_A = \frac{DD}{c_{GC} * CR_A} \qquad ACF_L = \frac{DD}{c_{GC} * CR_L}, \qquad \text{Eq. S1}$$

where c_{GC} is the dose calibration factor of the gamma camera. ACF_A and ACF_L were analyzed for all volunteers and all application modes.

^{81m}Kr-Gas Sinus Ventilation Imaging

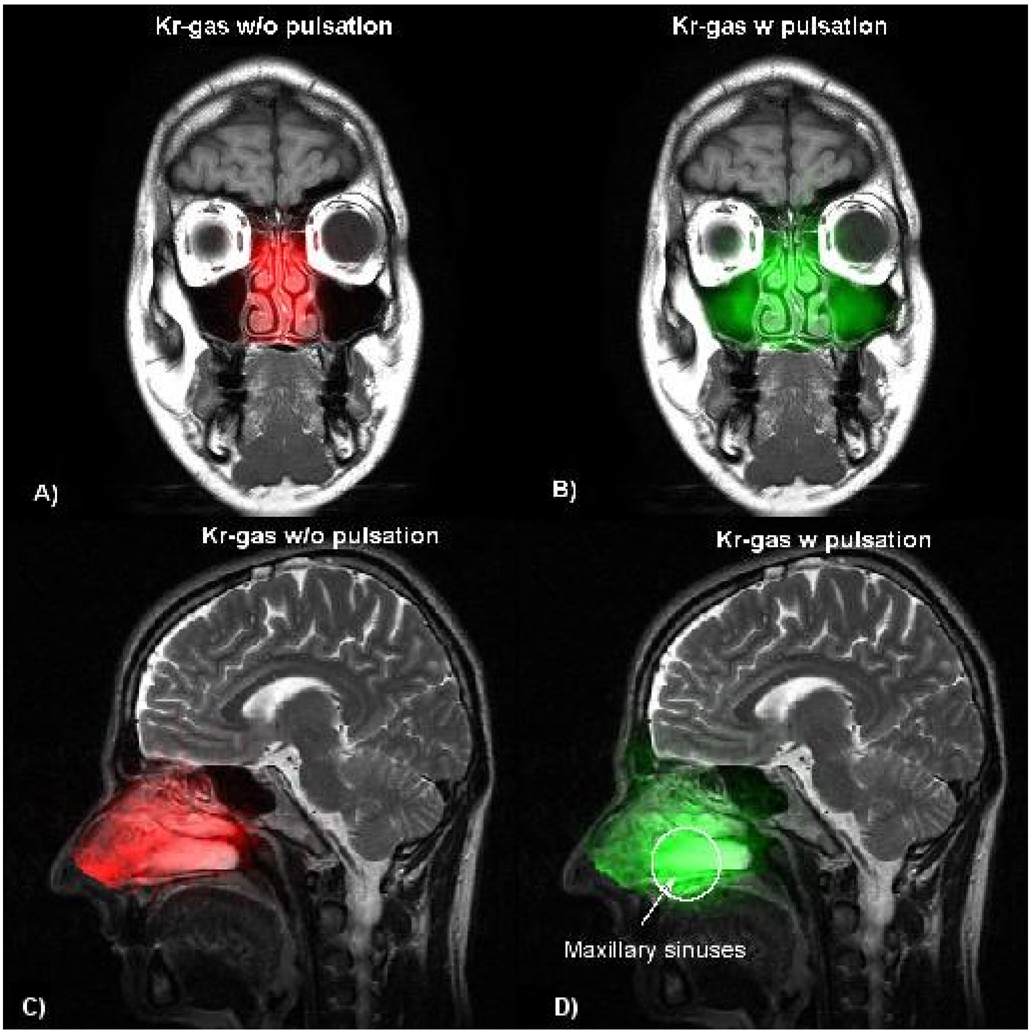

Figure S3: **Gamma camera imaging of ^{81m}Kr-gas ventilation of the nasal cavity and the sinuses.** Anterior (A and B) and lateral (C and D) imaging of ^{81m}Kr-gas ventilation of the nasal cavity and the sinuses without (A and C) and with (B and D) the vibration airflow technology (anterior and lateral gamma camera images superimposed onto coronal and sagittal MRI slices of the volunteer).

Case Study: Two Months Topical Steroid Aerosol Therapy Using Pulsating Aerosols

Three patients with CRS used the pulsating aerosol technique (PARI Sinus, PARI GmbH, Starnberg, Germany) to deliver once daily a steroid (Pulmicort, 1 mg/2ml) to the nasal cavities, as an alternative to FESS for two months. All nasal and sinus obstructions, which were apparent before the treatment, had disappeared after two months of therapy, as confirmed by fiber optic rhinoscopy and MRI (Figure S4). Sinus surgery could be avoided in these patients.

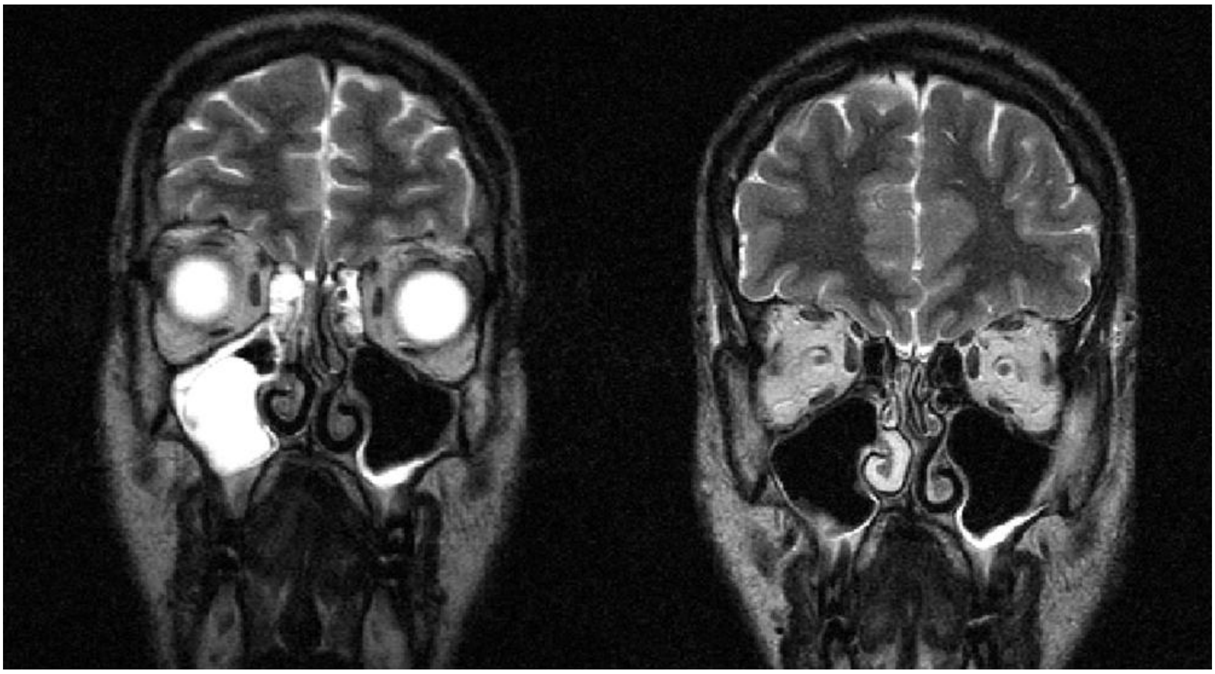

Figure S4: **MRI slices of a patient before and after topical steroid treatment using a pulsating aerosol device.** Coronal MRI (T2 weighted) slice of a patient before (left) and after (right) a two months once daily treatment with steroids (Pulmicort respules, 1 mg/2 mL) using the PARI Sinus pulsating aerosol device.
